# Breadstick fortification with red grape pomace: effect on nutritional, technological and sensory properties

**DOI:** 10.1002/jsfa.11596

**Published:** 2021-11-03

**Authors:** Giada Rainero, Federico Bianchi, Corrado Rizzi, Mariasole Cervini, Gianluca Giuberti, Barbara Simonato

**Affiliations:** ^1^ Department of Biotechnology University of Verona Verona Italy; ^2^ Department for Sustainable Food Process Università Cattolica del Sacro Cuore Piacenza Italy

**Keywords:** bakery products fortification, winemaking by‐products, rheological properties, phenolic compounds, sensory analysis

## Abstract

**BACKGROUND:**

Grape pomace (GP), a wine‐making by‐product rich in dietary fiber (DF) and total phenolic compounds (TPC), is a potential functional ingredient in the fortification of baked goods.

**RESULTS:**

In the present study, fortified breadsticks samples were obtained by replacing wheat flour with 0, 5 and 10 g 100 g^−1^ of powdered GP (GPP). The GPP inclusion affected the rheological properties of the doughs by increasing the water absorption and tenacity (*P*) at the same time as reducing the extensibility (*L*), with a significant increase in the *P*/*L* value and a decrease in the swelling index (*G*) value and deformation energy (*W*). Textural characteristics of breadsticks were influenced by the GPP addition, showing a reduction in hardness and fracturability as the amount of GPP increased in the recipe. The GPP fortified breadsticks exhibited decreased pH, volume and specific volume values compared to the control. The TPC and the antioxidant capacity increased in GPP fortified breadsticks, whereas the increased amount of DF allowed the products to benefit from the claim ‘high fiber content’ at the highest level of GPP inclusion. The sensory evaluation revealed that GPP addition increased wine odor, acidity, bitterness, astringency and hardness, and decreased the regularity of alveolation and friability. Finally, the GPP fortified products achieved good sensorial acceptability.

**CONCLUSION:**

GPP improved the nutritional values of fortified breadsticks and changed the rheology of dough and breadsticks' technological properties without affecting sensory acceptability. © 2021 The Authors. *Journal of The Science of Food and Agriculture* published by John Wiley & Sons Ltd on behalf of Society of Chemical Industry.

## INTRODUCTION

In recent years, ready‐to‐eat foods have grown popular among consumers as a result of their convenience of consumption, ease of preparation and storage. Unfortunately, these foods are often rich in fats and sugars. Otherwise, an increasing segment of consumers considers mainly the nutritional and health aspect during their food expenditure.^1^ In this context, bakery products, widely consumed worldwide, could represent great potential as carriers of functional ingredients^2^, ^3^ that improve the wholesome global characteristic of these foodstuffs.

Food waste is deemed as a possible source of bioactive molecules with beneficial properties. The FAO estimated that, every year, 1.3 billion tons of food are wasted, such that the 2030 Agenda for Sustainable Development aimed to reduce food loss by promoting a sustainable lifestyle.^4^ United Nations Member States set the target of dropping food waste by adopting specific measures to reduce food loss throughout the production chain.^5^ An alternative purpose of food by‐products is their incorporation in different food matrices, thus becoming functional food ingredients and a source of bioactive compounds.^6^, ^7^

The wine industry leads to a considerable amount of waste, including grape pomace (GP). One hectolitre of wine produces approximately 17 kg of GP,^7^ representing a valuable source of bioactive molecules, such as phenolic compounds and dietary fiber (DF). However, even if GP could be considered as a novel food, its extract has been approved by the European Food Safety Authority as a food dye in marmalades, drinks, sweets, ice creams and pharmaceutical products.^8^

Polyphenols play a crucial role in preventing a series of non‐communicable diseases.^9^ Additionally, DF helps prevent obesity, reduce blood cholesterol levels and improve intestinal transit of stool.^10^ Food fortification aims at increasing the DF amount and can contribute to achieving the correct daily intake (25–30 g per day).^11^ In this context, GP, used as a high added‐value ingredient in different food preparations, improves a final product's nutritional profile and increases its value.^12^ However, the addition of fibres and polyphenols in bakery products could impact severely on their technological aspects. For example, dietary fibres could alter the gluten network, competing with starch for water absorption and change the rheological properties of doughs and the texture of the final product.^6^, ^7^ Therefore, baked food production is an excellent opportunity to develop the ‘circular economy’ concept by exploiting agro‐industrial by‐products.

Breadsticks are traditional pencil‐shaped sticks of bread that have been rolled and baked and are are extensively consumed for their taste, crispiness and extended shelf‐life.^13^ In addition, breadstick is a food preparation that lends itself well to fortification to improve the overall nutritional profile, as demonstrated in previous studies.^14^, ^15^, ^16^

The present study aimed to investigate the effect of the breadstick fortification with powdered grape pomace (GPP), evaluating the changes in the rheological characteristics of GPP enriched doughs and the technological features of the final products. In addition, the research focused on nutritional aspects such as phenolic compounds, dietary fiber content, antioxidant capability, sensory analysis of control and fortified breadsticks.

## MATERIALS AND METHODS

### Raw materials and grape pomace powder preparation

Common wheat flours were kindly supplied by Macinazione Lendinara (Arcole, Verona, Italy). Wheat flour composition was the following: total carbohydrates 70 g 100 g^−1^, protein 11.5 g 100 g^−1^, fat 1.2 g 100 g^−1^, total dietary fiber 3 g 100 g^−1^ and ash 0.6 g 100 g^−1^. In addition, extra virgin olive oil, common salt, active dry yeast and wheat malt flour were bought in a local market.

GP (*Vitis vinifera* cv. Cabernet), obtained after alcoholic fermentation (kindly provided by Ripa della Volta, Verona, Italy), were dried in a vacuum oven (VD 115; Binder GmbH, Tuttlingen, Germany) at 40 °C and 30 kPa.

Stems and seeds were manually removed, and GP was milled and sieved to obtain a powder of particle size < 200 μm. The powder thus obtained was stored under a vacuum in the dark.

### Preparation of breadsticks

The breadsticks were produced at Panificio Zorzi (Brentino Belluno, Verona, Italy), replacing the common wheat flour with 0, 5 and 10 g 100 g^−1^ of GPP (w/w), obtaining BS0, BS5 and BS10, respectively. For the preparation of the BS0 dough, 8 kg of wheat flour mix, 380 g of extra virgin olive oil, 200 g of dry *Saccharomyces cerevisiae*, 150 g of salt and 40 g of wheat malted flour were mixed. For BS0, BS5 and BS10, 4.5, 4.7, and 5.0 L of water, respectively, was added to the doughs to obtain mixtures with similar workability characteristics.

The ingredients were mixed with a professional planetary kneading machine (Planetary Kneading; Sammic, Bergamo, Italy). The doughs were processed with an automatic sheeter to obtain breadsticks (Industrial Breadstick Machine; Prim Italia Srl, Milano, Italy). The breadsticks were automatically placed on stainless steel baking trays. After the leavening phase of 30 min at 32 °C, the breadsticks were cooked in an electric oven at 168 °C for 27 min, cooled to room temperature, and finally packaged in a transparent polypropylene film, each containing approximately 50 g of samples. The breadsticks were stored at room temperature in the dark.

### Rheology of dough

Alveograph, farinograph and amylograph analyses were performed on doughs prepared by mixing water and wheat flour replaced with 0, 5 and 10 g 100 g^−1^ of GPP (w/w), obtaining D0, D5 and D10, respectively.

An alveograph (Chopin Technologies, Villeneuve La Garenne, France) (AACC method 54–30) was used to record the following parameters: deformation energy (*W*), tenacity (*P*), dough extensibility (*L*), swelling index (*G*) and the curve configuration ratio (*P*/*L*). Dough mixing properties such as water absorption, stability, development time, degree of softening (12 min after maximum) and quality number were measured using a Brabender Farinograph (Brabender, Duisburg, Germany) (AACC method 54‐21.02). An amylograph (Brabender, Duisburg, Germany) (AACC method 22‐10) was used to analyse the start of gelatinization (°C), gelatinization maximum (AU) and gelatinization temperature (°C).

### Functional properties of wheat and composite flours

Water absorption capacity (WAC) and oil absorption capacity (OAC) of wheat flour replaced with 0, 5, and 10 g 100 g^−1^ of GPP (w/w) (FG0, FG5 and FG10) were determined according to Kaushal *et al*.^17^ with slight modification. Three grams of sample were dispersed with 25 mL of distilled water or corn oil for WAC and OAC analysis, respectively. Samples were stirred periodically within 30 min and centrifugated for 25 min at 3000 × *g*. Supernatants were removed and pellets were weighed. The WAC and OAC were expressed as gram of water or oil 100 g^–1^ sample.

The water solubility index (WSI) was determined following the procedure for WAC, but supernatants were decanted into Petri plate and dried at 105 °C until achieving constant weight. WSI was expressed as:

$$\mathrm{WSI}=\frac{\text{weight of dried supernatants}}{\text{weight of sample}}\times 100$$

### Proximal composition of grape pomace powder and breadsticks

GPP and BS0, BS5 and BS10 were analyzed (AOAC 2000) for dry matter (DM, method 930.15), ash (method 942.05), crude protein (method 976.05), crude lipid (method 954.02), and total starch (method 996.11). Free sugars were assessed using the Megazyme assay kit K‐SUFRG 06/14 (Megazyme, Wicklow, Ireland). The total, soluble and insoluble dietary fiber (TDF, SDF and IDF, respectively) content was assessed enzymatically (Megazyme assay kit K‐TDFR‐200A).

### Technological characteristics of breadsticks

#### Moisture content, water activity, pH, volume and specific volume

The moisture content of the breadsticks was measured by the AACC method 44‐15A and the water activity (*a*_*w*_) with a Hygropalm HC2AW‐meter (Rotronic Italia, Milano, Italy) at 23 °C. The pH value was determined with a pH meter (Mettler‐Toledo Inc., Columbus, OH, USA) by mixing 4 g of minced sample with 20 mL of water. The specific volume (cm^3^ g^−1^) was determined by seed displacement (AACC method 10‐05.01).

#### Texture analysis

Texture characteristics in terms of hardness and fracturability were analyzed using a TA‐XT2i Texture Analyser (Stable Micro Systems, Godalming, UK) equipped with a three‐point bending rig (Part. No. HDP/3 PB). The analysis was performed at 1 mm s^−1^ initial speed and 3 mm s^−1^ test speed, with a 50‐g trigger force and a 5‐kg load cell. The maximum force was recorded as the hardness value, and the distance at the point of break was the fracturability value. Ten measurements for each sample were carried out.

#### Color analysis

The color of breadsticks was determined with a reflectance colorimeter (Chroma Meter CR‐300; Minolta, Osaka, Japan; Japan illuminant D65) following the CIE L* a* b* color system. The lightness (L*) and color parameters (+*a*: red −*a*: green; +*b*: yellow; −*b*: blue) were assessed. The total color difference between samples was calculated using:

$$∆E=\sqrt{∆L^{2}+∆a^{2}+∆b^{2}}$$

$$∆L=\left(L-L_{0}\right);∆a=\left(a-a_{0}\right);∆b=\left(b-b_{0}\right)$$

where *L*, *a,* and *b* are the parameters of fortified breadsticks (BS5 and BS10) and *L*_0_, *a*_0_, and *b*_0_ are the values of the control breadstick (BS0).

### Total phenol content (TPC) and antioxidant capacity

Five hundred milligrams of powdered breadsticks or GPP were incubated with 7.5 mL of MeOH:HCl 97:3 (v/v) for 16 h in the dark at room temperature.^18^ Supernatants were collected after centrifugation (3500 × *g* for 10 min) and used for TPC, 2,2′‐azino‐bis (3‐ethylbenzothiazoline‐6‐sulfonic acid (ABTS) and ferric reducing ability of plasma (FRAP) radical scavenging activities determination, as described by Tolve *et al*.^7^

### Sensory evaluation of breadsticks

The sensory profile of breadsticks was evaluated by a group of 18 trained panellists (8 men, 10 women, 22–28 years old), recruited from voluntary students of the Department of Biotechnology of the University of Verona. After the generation of 14 sensory attributes (i.e. color uniformity, alveolation regularity, fragrance, wine odor, global odor, global flavor, sweetness, saltiness, acidity, bitterness, friability, hardness, grittiness and astringency), judges were trained to recognize their intensities. The judges received two breadsticks placed on a covered plate in a balanced and randomized order. A nine‐point scale was used to describe the intensity of all attributes, with 1 representing the lowest intensity and 9 indicating the highest. Panellists also commented on the overall acceptability of breadsticks: samples were considered acceptable if their mean scores were > 5 (neither like, nor dislike).

### Statistical analysis

All data represent the means of at least three measures, and results are reported as the mean ± SD. The comparison of means was conducted using the analysis of variance (ANOVA) with a post‐hoc Tukey's test ( *p* < 0.05). Statistical analyses were performed using XLSTAT (Addinsoft SARL, Paris, France).

## RESULTS AND DISCUSSION

### Rheological properties

The rheological properties of doughs were influenced (*p* < 0.05) by the addition of GPP. The farinograph characteristics of control dough (D0) and doughs with added GPP (D5 and D10) are reported in Table 1. Water absorption, the amount of water required for the dough to have a definite consistency of 500 UB, increased significantly (*p* < 0.05) from 58.93% in the control dough to 59.97% in the D10 dough. This behaviour is probably associated with the ability of GPP fibers to retain water in the matrix. The DF structure contains a significant number of hydroxyl groups that can establish hydrogen bonds with water molecules. This agrees with other studies investigating the effect of the addition of different types of fibers in wheat flour doughs.^19^, ^20^

**Table 1:** Rheological characteristics of the doughs supplemented with 0, 5 and 10 g 100 g^−1^ of grape pomace powder (D0, D5 and D10, respectively)

Farinograph	D0	D5	D10
Water absorption (%)	58.93 ± 0.15 a	59.17 ± 0.23 a	59.97 ± 0.29 b
Stability (min)	9.93 ± 0.71 a	7.57 ± 1.00 ab	7.03 ± 0.84 b
Development time (min)	2.83 ± 1.29 a	5.63 ± 0.12 a	3.40 ± 1.66 a
Degree of softening (UB)	43.33 ± 7.51 a	62.67 ± 2.52 ab	67.33 ± 9.02 b
Quality number	108.33 ± 7.51 a	88.00 ± 4.36 ab	79.67 ± 9.07 b

Alveograph	D0	D5	D10
*P* (mm)	96.33 ± 4.62 a	157.33 ± 2.08 b	215.33 ± 6.66 c
*L* (mm)	106.33 ± 6.51 a	51.00 ± 2.65 b	25.33 ± 2.31 c
*P*/*L*	0.91 ± 0.09 a	3.09 ± 0.19 b	8.53 ± 0.59 c
*G* (cm^3^)	22.97 ± 0.70 a	15.90 ± 0.44 b	11.20 ± 0.52 c
*W* (10^−4^ J)	321.67 ± 7.23 a	319.00 ± 12.17 a	244.00 ± 26.96 b

Amylograph	D0	D5	D10
Start of gelatinization (°C)	63.00 ± 0.00 a	64.50 ± 0.00 ab	65.00 ± 0.87 b
Gelatinization maximum (AU)	1525.00 ± 8.66 a	1791.67 ± 23.63 b	1996.67 ± 24.66 c
Gelatinization temperature (°C)	94.90 ± 0.62 a	94.30 ± 0.35 a	94.60 ± 0.75 a

These values are the mean ± SD of three independent experiments. The comparison of means was conducted using ANOVA with a post‐hoc Tukey's test at *p* < 0.05. Data with different lowercase letters in each line are significantly different.

The time stability of doughs, which is related to the protein content of the wheat flour, and the development of the gluten network,^21^ decreased (*p* < 0.05) as GPP increased in the recipe, in line with previous findings.^20^ Replacing wheat flour with GPP led to a dilution of gluten content of the flour blend, causing a significant reduction (*p* < 0.05) in the stability time in D10 compared to D0. This result contrasts with the outcomes reported by Tolve *et al*.^7^ concerning the time stability of bread fortified with GPP, which progressively increased with the fortification. By contrast, the same trend was observed by Mironeasa *et al*.,^22^ where the stability time of doughs fortified with 5 and 9 g 100 g^−1^ (w/w) of GP flour with a particle size < 200 μm showed a stability of 7.40 min, similar to BS5 and BS10 samples.

The degree of softening increased (*p* < 0.05) in D10 compared to D0. A similar result was obtained by Šporin *et al*.^23^ in doughs added with Zelen GP, probably as a result of the reduction in gluten content and destruction of the gluten network by GPP fibers. The same study revealed a reduction in the quality number of doughs, as observed for D5 and D10 samples. Development time was not affected by the inclusion of GPP in doughs, showing an average of 3.95 min.

The alveograph results are summarized in Table 1. The *P* value, also known as tenacity, is an indicator of doughs' capacity to retain gas, whereas the *L* value, also called elasticity, reveals the extension capacity of the doughs without breakdown. The *P*/*L* ratio on the control dough was 0.91; this ratio, for a good bakery, should not exceed 2.^24^ The addition of GPP in the doughs caused an increase (*p* < 0.05) in the *P* values, ranging from 96.33 to 215.33 mm, and a decrease (*p* < 0.05) in the *L* values, from 106.33 to 25.33 mm, for D0 and D10, respectively. As a result, a higher (*p* < 0.05) *P*/*L* ratio was measured in D5 and D10 compared to the control (D0). The interaction between wheat flour proteins and the increased amount of fibers from GPP may explain the increase in *P* value.^22^ In addition, D5 and D10 samples were more tenacious and less extensible than D0 (*p* < 0.05), as already outlined in GPP fortified bread.^7^ These results could be ascribed to the rigid nature of DF that increases the doughs tenacity and to the partial replacement of wheat flour with GPP, hence the reduction of the gluten protein content that hindered the formation of a strong gluten structure.^25^ Moreover, the GPP fiber components could compete with gluten proteins for water absorption during kneading, thus forming a weakened gluten network.

The *G* value, also named the swelling index, is described by the size of the bubble after air insufflation. The *G* value of the control sample was higher (*p* < 0.05) than those recorded for fortified samples, being 22.97 *versus* 15.90 and 11.20 cm^3^ for D0, D5 and D10, respectively. This is related to the formation of a very tenacious and not very extendable dough, as detected by *P* and *L* values of the fortified doughs. For the same reason, deformation energy (*W*), defined as the area under the alveogram curve, decreased (*p* < 0.05) in D10 (but not for D5) compared to D0. Similar results were reported for doughs with barley husk and purple sweet potato flour.^26^, ^27^

The inclusion of GPP modified the gelatinization characteristics of the doughs (Table 1). GPP inclusion levels influenced the temperature of the beginning gelatinization, which increased from 63.0 to 65.0 in D0 and D10, respectively (*p* < 0.05). This could be related to the presence of GPP fibers that, competing with the starch granules for water absorption, slowed down or limited starch gelatinization, which starts at a higher temperature.^22^ In addition, the GPP inclusion significantly affected (*p* < 0.05) the gelatinization maximum of the dough, identified as the maximum viscosity reached, which increased in D5 and D10 doughs compared to D0 dough, as reported previously.^7^, ^22^ The rise in temperature during pasting may cause the formation of polymeric complexes as a consequence of fibers' interactions with low molecular weight amylopectin and amylopectin molecules^28^ that can justify the increase in viscosity. Finally, gelatinization temperature was not affected by the addition of GPP, being on average 94.6 °C (*p* > 0.05).

### Functional properties of wheat and composite flours

The WAC, OAC and WSI of flour samples FG0, FG5 and FG10 (flour substituted by 0, 5, and 10 g 100 g^−1^ of GPP powder, respectively) are summarized in Table 2. In particular, the WAC is an indicator of the flour functionality for binding and holding water. WAC decreased significantly (*p* < 0.05) from 63.35 to 57.18 with the inclusion level of GPP, without significant differences among FG5 and FG10. As described by Simsek and Süfer^29^ and Gull *et al*.,^30^ a higher WAC in FG0 could be ascribed to wheat flour components leaching and a modification of starch granules structure.

**Table 2:** Water binding capacity (WAC), oil binding capacity (OAC), and water solubility index (WSI) of wheat flour supplemented with 0, 5 and 10 g 100 g^−1^ of grape pomace powder (FG0, FG5 and FG10, respectively)

Sample	FG0	FG5	FG10
WAC (g 100 g^−1^)	63.35 ± 1.33 a	57.18 ± 2.07 b	57.99 ± 1.19 b
OAC (g 100 g^−1^)	73.06 ± 1.19 a	74.04 ± 0.38 a	75.69 ± 0.53 b
WSI (%)	5.32 ± 0.02 a	8.54 ± 0.32 b	9.44 ± 0.09 c

These values are the mean ± SD of three independent experiments. The comparison of means was conducted using ANOVA with a post‐hoc Tukey's test at *p* < 0.05. Data with different lowercase letters in each line are significantly different.

The OAC is the measurement of the oil holding in the capillaries of the flour particle through physical entrapment of oil within the protein structure and non‐covalent interactions such as hydrophobic, electrostatic and hydrogen bindings among oil and proteins.^31^ This parameter is of great importance since fats act as flavor retainers and increase the mouthfeel of the foods. As reported in Table 2, the FG10 was characterized by the highest OAC among the other flour mixes, being 75.69 g 100 g^−1^ (*p* < 0.05). The possible explanation for the OAC increase in FG10 could be related to different content in the protein non‐polar side chains that might bind the hydrocarbon side chains of the oil.^17^ However, because fibers showed a good oil quality absorption,^29^, ^32^ we cannot exclude their contribution in influencing the OAC in the flour.

The WSI describes the solubility of flours in water, thus indicating the difference of soluble molecules in flour or flour blends.^33^ The WSI increased gradually from 5.32 g 100 g^−1^ in FG0 to 9.44 g 100 g^−1^ in FG10 composite flour (*p* < 0.05). The increase in WSI following the addition of GPP indicates that fortified flour had enhanced the quantity of soluble materials such as soluble dietary fibers.^17^

### Chemical characterization of grape pomace and breadsticks

The chemical composition of GPP, control and GPP‐fortified breadsticks is reported in Table 3. The ash content increased with increasing levels of GPP in the formulation, ranging from 2.50 to 3.08 g 100 g^−1^ DM for BS10 and BS0, respectively (*p* < 0.05). The ash content depends in part on macro‐and micro‐elements contained in GPP, especially K, P, Mn, Fe and Zn.^34^ The replacement of wheat flour with different amounts of GPP caused a reduction in crude protein and total starch contents in BS5 and BS10 samples compared to BS0 (*p* < 0.05). The inclusion of GPP in the recipe caused a significant rise (*p* < 0.05) in the total DF content, ranging from 3.47 to 5.81 and 8.55 g 100 g^−1^ DM for BS0, BS5 and BS10, respectively. Consequently, the BS10 sample can be claimed as a ‘high fibers content’ food product because it contains more than 6 g 100 g^−1^ of total dietary fiber. A similar DF level was observed in other studies producing bread and cookies with grape skin powder.^35^, ^36^, ^37^ From a nutritional perspective, the production of cereal‐based foods rich in fibers can be an aid to reach the recommended daily intake of approximately 30 g per day for adult humans.^11^ In addition, the most relevant portion of total DF in GPP fortified breadsticks was represented by the insoluble dietary fiber fraction (approximately 90% of the total), which leads to rapid gastric emptying, decreased intestinal transit time and increased faecal mass, thus promoting digestive regularity.^10^ Lastly, the lipid and free sugar contents did not vary in all samples, being on average 4.68 and 0.09 g 100 g^−1^ (*p* > 0.05), respectively.

**Table 3:** Proximate composition (g 100 g^−1^) of grape pomace powder (GPP), control breadsticks (BS0) and breadsticks fortified with 5 and 10 g 100 g^−1^ of GPP (BS5 and BS10)

Proximate composition	GPP	BS0	BS5	BS10
Crude lipid	4.38 ± 0.15	4.59 ± 0.18 a	4.62 ± 0.21 a	4.77 ± 0.15 a
Crude protein	13.86 ± 0.10	13.63 ± 0.10 a	12.57 ± 0.07 b	12.46 ± 0.12 b
Total starch	–	70.96 ± 0.85 a	67.78 ± 0.25 b	65.71 ± 1.06 c
Total dietary fiber	57.02 ± 0.17	3.47 ± 0.13 a	5.81 ± 0.15 b	8.55 ± 0.22 c
Insoluble fiber	52.18 ± 0.20	2.23 ± 0.15 a	4.07 ± 0.12 b	6.27 ± 0.23 c
Ash	9.92 ± 0.07	2.50 ± 0.00 a	2.82 ± 0.04 b	3.08 ± 0.04 c
Free sugars	–	0.099 ± 0.002 a	0.098 ± 0.004 a	0.100 ± 0.004 a

These values are the mean ± SD of three independent experiments. The comparison of means was conducted using ANOVA with a post‐hoc Tukey's test at *p* < 0.05. Data with different lowercase letters in each line are significantly different.

### Physiochemical characterization of GPP and breadsticks and textural properties of breadsticks

**Figure 1:**
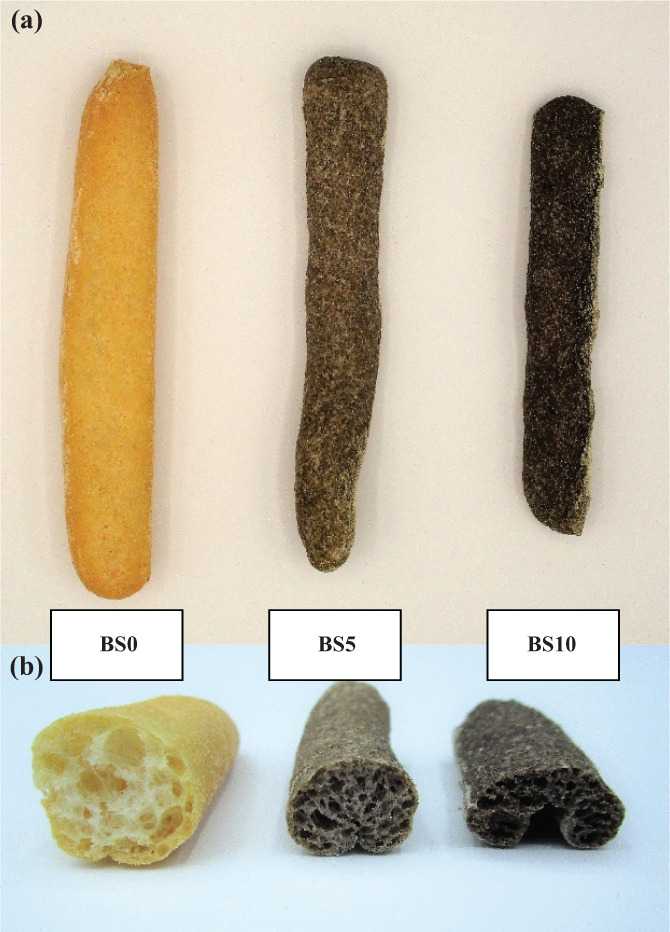
Breadsticks (a) and sections (b) of control breadsticks (BS0) and breadsticks fortified with 5 and 10 g 100 g^−1^ of grape pomace powder (BS5 and BS10).

The moisture content, *a*_*w*_ and pH values of GPP were 6.39 ± 0.04, 0.34 ± 0.003 and 3.35 ± 0.02. Table 4 shows the physicochemical values and the technological properties of BS0, BS5 and BS10. The *a*_*w*_ of samples showed no statistical differences after GPP fortification, resulting in an average of 0.188 ± 0.004. The pH values decreased significantly (*p* < 0.05) in the GPP fortified samples compared to the control, being on average 4.37 *versus* 5.43, respectively. The acidic conditions could lead to more tenacious and less extensible doughs,^38^ as indicated by the rheological results, causing a reduction of volume and specific volume of fortified breadsticks compared to the control (*p* < 0.05) (Fig. 1b). This behaviour was previously described in fortified bread.^7^, ^39^

**Table 4:** Physiochemical characterization, textural properties and color, expressed as L* (lightness), a* (red/green), b* (blue/yellow), and ∆*E* (total color difference) of control breadsticks (BS0) and breadsticks fortified with 5 and 10 g 100 g^−1^ of grape pomace powder (BS5 and BS10)

Physiochemical characterization	BS0	BS5	BS10
Moisture content (g 100 g^−1^)	2.59 ± 0.12 a	2.81 ± 0.13 a	3.06 ± 0.01b
pH	5.43 ± 0.01 a	4.56 ± 0.02 b	4.18 ± 0.02c
*a*_*w*_	0.185 ± 0.01 a	0.192 ± 0.01 a	0.188 ± 0.01 a
Volume (cm^3^)	77.31 ± 2.29 a	47.96 ± 1.89 b	37.76 ± 1.61 c
Specific volume (cm^3^ g^−1^)	3.82 ± 0.11 a	2.43 ± 0.12 b	1.82 ± 0.04c

Texture properties	BS0	BS5	BS10
Hardness (N)	1822.54 ± 394.02 a	949.71 ± 367.63 b	746.29 ± 294.58 b
Fracturability (N)	1.11 ± 0.52 a	0.31 ± 0.19 b	0.32 ± 0.15 b

Color values	BS0	BS5	BS10
L*	92.22 ± 0.40 a	69.98 ± 0.66 b	65.02 ± 0.73 c
a*	−2.06 ± 0.19 a	1.54 ± 0.39 b	3.21 ± 1.37 b
b*	18.29 ± 1.37 a	11.31 ± 0.32 b	8.58 ± 1.30 c

Color differences	BS0 – BS5	BS0 – BS10	BS5 – BS10
∆*E*	23.59	29.36	5.9

These values are the mean ± SD of three independent experiments. The comparison of means was conducted using ANOVA with a post‐hoc Tukey's test at *p* < 0.05. Data with different lowercase letters in each line are significantly different.

The reduction of volume is also highly correlated with the fiber content of breadsticks (*r* = −0.944). The GPP fibers modified the rheological properties of the doughs by changing their leavening and aeration properties. According to Fu *et al*.,^40^ DF may alter the gluten network as a result of a rigid structure and, for the dilution of gluten proteins, compromise the ability of doughs to hold the air bubble during leavening. BS5 and BS10 samples were flatter than BS0 and without the typical swollen and well‐ventilated structure of leavened bakery products. A similar result has been observed in breadsticks containing 25 and 35 g 100 g^−1^ of brewer's spent grain, a fiber‐rich by‐product of the beer industry.^41^

The texture is one of the most critical quality attributes in breadsticks because consumers appreciate a crisp and crunchy texture. As a result of the addition of GPP in the recipe, both the hardness and fracturability of breadsticks decreased (*p* < 0.05) compared to BS0, but without differences between BS5 and BS10 samples (Table 4). A similar behaviour relating to the hardness decrement in breadsticks fortified with olive pomace was described by Simsek and Süfer.^29^ BS0 showed a higher strength value at breakage (*p* < 0.05). This may indicate a more solid and firm gluten network because it also had a higher protein content. The dilution of gluten content as a resut of GPP fortification may produce softer breadsticks, as reported by Petchoo *et al*.^16^ for breadsticks with resistant starch added.

In addition, a damaged and unconstructed gluten protein network may cause a decrease in fracturability in GPP‐fortified samples. Similar results were reported for breadsticks fortified with brewer's spent grain and germinated or non‐germinated legumes and for cookies with white grape pomace and fiber from Chiku.^13^, ^41^, ^42^, ^43^

### Color analysis

The color of breadsticks was influenced (*p* < 0.05) by the addition of GPP, as detailed in Table 4 and as observable in Fig. 1(a). Because of the dark color of GPP, the lightness significantly decreased in BS5 and BS10 (*P* < 0.05). Regarding color parameters, the a* value increased (*p* < 0.05), whereas the b* value decreased (*p* < 0.05) because of fortification. Similarly, these color changes involved bread and pasta samples with red grape pomace.^7^, ^23^, ^44^ By contrast, a decrease in a* and an increase in b* values was detected in cakes produced with growing amounts of red grape pomace.^45^ In muffins and biscuits fortified with red grape pomace, a decrease in all color parameters was observed instead.^36^, ^46^ Finally, the total color difference generally used to describe the color variation was more noticeable between BS0 and fortified samples than the comparison between BS5 and BS10.

### TPC and antioxidant capacity

The total phenol content of GPP used was 18.34 ± 0.42 mg GAE g^−1^ DM, whereas the antioxidant activity, evaluated with FRAP and ABTS, was 149.27 ± 7.73 and 104.73 ± 2.80 μm TE g^−1^ DM, respectively. The TPC and the antioxidant activity of breadsticks were influenced by the GPP addition level (*p* < 0.05), with high correlation coefficients between them (*r* = 0.967 TPC *versus* ABTS and *r* = 0.958 TPC *versus* FRAP) (Table 5). In particular, the TPC increased from 72.21 to 171.83 mg GAE 100 g^−1^ DM and the FRAP increased from 360.60 to 2801.00 μm TE 100 g^−1^ DM for BS0 and BS10, respectively (*p* < 0.05). A similar TPC value was detected in bread and pasta with the same amount of GPP added^7^, ^44^ and in bread enriched with 10 and 15 g 100 g^−1^ of wine grape pomace.^37^ Muffin fortified with 5 and 10 g 100 g^−1^ of GPP presented comparable antioxidant capacity values.^37^ In any case, the TPC and antioxidant activity of grape flour depends mainly on the grape variety, the presence/absence of seeds, and the drying technique.^23^

**Table 5:** Total phenolic compounds (TPC) and antioxidant activity (FRAP and ABTS) of control breadsticks (BS0) and breadsticks fortified with 5 and 10 g 100 g^−1^ of grape pomace powder (BS5 and BS10)

Sample	BS0	BS5	BS10
TPC (mg GAE 100 g^−1^ DM)	72.21 ± 6.77 a	124.54 ± 12.68 b	171.83 ± 11.36 c
FRAP (μm TE 100 g^−1^ DM)	360.60 ± 47.86 a	1962.94 ± 210.39 b	2801.00 ± 258.66 c
ABTS (μm TE 100 g^−1^ DM)	233.53 ± 23.26 a	701.06 ± 58.42 b	1139.25 ± 27.17 c

These values are the mean ± SD of three independent experiments. The comparison of means was conducted using ANOVA with a post‐hoc Tukey's test at *p* < 0.05. Data with different lowercase letters in each line are significantly different.

### Sensory evaluation

**Figure 2:**
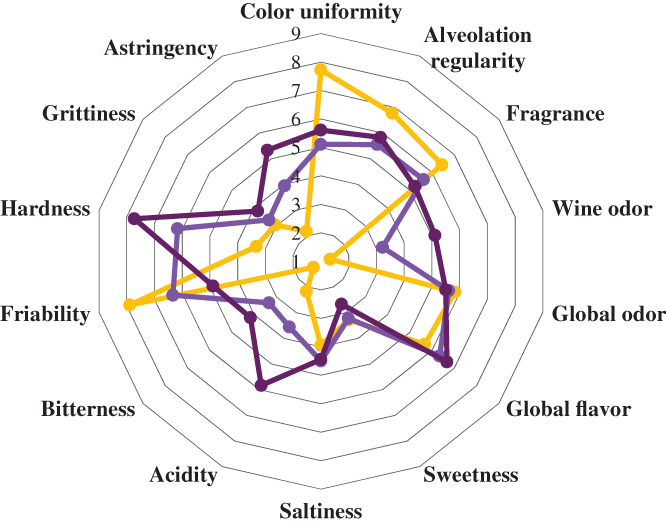
Sensory score of quality attributes of control breadsticks (BS0 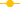 ) and breadsticks fortified with 5 and 10 g 100 g^−1^ of grape pomace powder (BS5 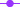 and BS10 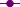).

The fortification process of breadsticks with GPP caused variation in some parameters chosen for sensory analysis (Fig. 2). Visually, BS0 showed a better color uniformity and a more regular alveolate structure than fortified samples. From the olfactory point of view, as the amount of GPP added increased, the wine odor of fortified samples had enhanced markedly, as previously observed for pasta and bread with the addition of grape pomace.^7^, ^44^ By contrast, the fragrance and global odor were not affected by the presence of GPP in the samples. The taste sensations most influenced by the fortification were acid and bitterness. In particular, the acid was significantly perceived more in BS10 than in BS5. The acidic taste also increased in bread fortified with pomace, as reported by Šporin *et al*. and Tolve *et al*.^7^, ^23^ Fortified breadsticks were more bitter compared to the control sample, as reported by Theagarajan *et al*.,^47^ in cookies with an increase in the amount of grape pomace in the dough. On the other hand, there were no differences in global flavor, sweetness and saltiness. Tactile sensations such as friability and hardness of the breadsticks were affected in reverse because of the fortification process. The friability of the samples gradually decreased with the amount of GPP added. By contrast to that found in mechanical texture analysis (Table 4), the hardness increased significantly in the fortified samples compared to the control, especially in BS10. Nevertheless, Petchoo *et al*.^16^ reported the same issue in breadsticks enriched with resistant starch. In addition, probably due to tannins commonly contained in grapes, astringency was significantly enhanced in BS5 and BS10.^48^ Finally, the overall acceptability was similar between BS0 and BS5, with an average of 7.39 ± 1.04 and 6.94 ± 1.26, respectively, whereas BS10 got a lower overall acceptability rating, with an average of 5.89 ± 1.37. However, these values exceeded the minimum threshold, set at 5.

## CONCLUSIONS

The results of the present study highlight the significant impact of GPP addition on the properties of doughs and breadsticks as final products. The replacement of wheat flour by GPP produced more tenacious and less extensible doughs than the control sample, with a significant increase in the *P*/*L* value and a decrease in the *G* value and deformation energy (*W*). Changes in the rheological characteristics of doughs have led to the production of fortified samples of lower volume and specific volume than the control sample. In addition, the inclusion of GPP caused acidification of the breadsticks and substantially influenced their color and texture characteristics. Nutritionally, the GPP fortified breadsticks had a higher content of phenolic compounds and dietary fiber. BS10 can benefit from the claim ‘high fiber’ content because it contains more than 6 g 100 g^−1^ of dietary fiber. In addition, despite fortification with GPP influencing most of the descriptors chosen for sensory analysis, fortified breadsticks showed good overall acceptability. Based on these results, GPP has proved to be a valuable functional ingredient for producing breadsticks rich in fiber and antioxidants and with a good sensory acceptability.

## CONFLICT OF INTERESTS

The authors declare that they have no conflicts of interest.
